# Deep Learning Model for Prediction of Progressive Mild Cognitive Impairment to Alzheimer’s Disease Using Structural MRI

**DOI:** 10.3389/fnagi.2022.876202

**Published:** 2022-06-02

**Authors:** Bing Yan Lim, Khin Wee Lai, Khairunnisa Haiskin, K. A. Saneera Hemantha Kulathilake, Zhi Chao Ong, Yan Chai Hum, Samiappan Dhanalakshmi, Xiang Wu, Xiaowei Zuo

**Affiliations:** ^1^Department of Biomedical Engineering, Faculty of Engineering, Universiti Malaya, Kuala Lumpur, Malaysia; ^2^Department of Computing, Faculty of Applied Sciences, Rajarata University of Sri Lanka, Anuradhapura, Sri Lanka; ^3^Department of Mechanical Engineering, Universiti Malaya, Kuala Lumpur, Malaysia; ^4^Department of Mechatronics and Biomedical Engineering, Lee Kong Chian Faculty of Engineering and Science, Universiti Tunku Abdul Rahman, Petaling Jaya, Malaysia; ^5^Department of Electronics and Communication Engineering, Faculty of Engineering and Technology, College of Engineering and Technology, SRM Institute of Science and Technology, Chennai, India; ^6^School of Medical Information & Engineering, Xuzhou Medical University, Xuzhou, China; ^7^Department of Psychiatry, The Affiliated Xuzhou Oriental Hospital of Xuzhou Medical University, Xuzhou, China

**Keywords:** Alzheimer’s disease, deep learning, prediction, magnetic resonance imaging, mild cognitive impairment

## Abstract

Alzheimer’s disease (AD) is an irreversible neurological disorder that affects the vast majority of dementia cases, leading patients to experience gradual memory loss and cognitive function decline. Despite the lack of a cure, early detection of Alzheimer’s disease permits the provision of preventive medication to slow the disease’s progression. The objective of this project is to develop a computer-aided method based on a deep learning model to distinguish Alzheimer’s disease (AD) from cognitively normal and its early stage, mild cognitive impairment (MCI), by just using structural MRI (sMRI). To attain this purpose, we proposed a multiclass classification method based on 3D T1-weight brain sMRI images from the ADNI database. Axial brain images were extracted from 3D MRI and fed into the convolutional neural network (CNN) for multiclass classification. Three separate models were tested: a CNN built from scratch, VGG-16, and ResNet-50. As a feature extractor, the VGG-16 and ResNet-50 convolutional bases trained on the ImageNet dataset were employed. To achieve classification, a new densely connected classifier was implemented on top of the convolutional bases.

## Introduction

Alzheimer’s disease (AD) is a progressive disease that causes neuronal loss and dementia in the elderly. Alzheimer’s disease patients typically exhibit progressive memory loss at the outset, followed by cognitive decline and, eventually, loss of independence. It is predicted that by 2050, one out of every 85 people in the world will have AD (Brookmeyer et al., 2007). At the moment, there are approximately 90 million people who have been identified as having AD, and the number of diseased patients is expected to reach 300 million by 2050 (Zhu et al., 2015).

There are medications that can provide temporary moderate symptom relief or slow the progression of AD, and these treatments have been shown to help patients with AD by achieving maximum cognitive function and maintaining independence for a period of time. However, there are currently no effective or safe drugs or therapies for curing Alzheimer’s disease or altering the disease process in the brain (Tatiparti et al., 2020). The search for effective strategies to treat or prevent Alzheimer’s disease remains one of the most difficult endeavors in medicine. As a result, it is critical to detect Alzheimer’s disease in its early or prodromal stages so that patients can receive treatment before the disease progresses. Currently, the standard non-invasive clinical strategy for performing prognostic prediction for Alzheimer’s disease is manual assessment *via* structural neuroimaging such as magnetic resonance imaging (MRI) or computed tomography (CT). Computer-aided methods based on artificial intelligence (AI) algorithms are currently being used to accomplish AD diagnostics (Wen et al., 2020).

In tandem with the rapid growth of AI, academics have been employing AI techniques such as deep learning to address complex problems in a variety of sectors, particularly medicine. Researchers have extended the use of multiple deep learning models to diagnose various stages of Alzheimer’s disease. Current neuroimaging investigations that use computer-aided system studies have made substantial progress in classifying Alzheimer’s disease (AD) and cognitively normal (CN) participants. Even though the binary classification of AD and CN participants performed admirably, it is not as useful as predicting the early-stage change of moderate cognitive impairment (MCI) to AD. The majority of research stopped at a binary categorization, without predicting whether a patient had MCI or the likelihood of converting to AD.

Detecting Alzheimer’s disease in its prodromal stage, or anticipating its potential, is critical for its treatment, just as it is for other diseases. Treatments are successful if AD patients receive them as soon as feasible after being suspected of having AD biomarkers or symptoms. A 1-year delay in the progression of Alzheimer’s disease can decrease the number of afflicted people by 10% (McKhann et al., 2011). According to the statistics, detecting Alzheimer’s disease in its early stages is critical to reduce the number of patients worldwide.

Neurologists must manually study brain scans and undertake cognitive assessments during the diagnosis of Alzheimer’s disease in order to make an accurate diagnosis of the symptoms and course of the disease. Because subtle changes in brain anatomy can be observed years before distinct biomarkers can be visualized by humans, it is realized that the human visual system is incapable of identifying subtle changes in underlying brain structure that may contain vital information about a patient’s disease state, even when the analysis is performed by the experienced neurologists. As a result, an AI-based computer-aided system can assist neurologists in detecting complicated brain illnesses while reducing the potential for misdiagnosis. Moreover, it is expected to decrease the workload on medical professionals and reduce the frequency of patient visit and waiting time. Many recent studies (Basaia et al., 2019; Bi et al., 2020; Jiang et al., 2020; Guo et al., 2021; Hett et al., 2021; Mehdipour Ghazi et al., 2021; Deng et al., 2022) have been conducted to forecast early stages of Alzheimer’s disease. The goal of this study is to build a computer-aided system based on a deep learning algorithm to evaluate the pathological brain structural changes in MRI data in order to forecast the early stages of Alzheimer’s disease before it progresses to the severe stages. The contributions of this proposed study are as follows:

1. Performing novel preprocessing procedures on brain structural MRI used for training and testing the convolutional neural network.
2. Implementing CNN to perform multiclass classification (3-way) to classify cognitively normal (CN), MCI, and AD subjects.
3. Evaluating the performance by metrics such as accuracy, precision, recall, and F1-score.

The rest of this article is organized as follows: the following section discusses the materials and methods used in this study and the subsequent section elaborates the experimental results and discussion. The final section emphasizes the conclusion and future research directions.

## Materials and Methods

### Dataset—Alzheimer’s Disease Neuroimaging Initiative

The relevant data were retrieved from the database of the Alzheimer’s Disease Neuroimaging Initiative (ADNI), which is available publicly upon approval from the ADNI. The ADNI database contains multiple collections of MRI images categorized by phase of the study, for example, ADNI1, ADNI2, ADNI-GO, and ADNI3 (as of August 2021). This study adopts all the sMRI data in the ADNI1 collection. A total of 819 subjects (229 CN, 398 with MCI, and 192 with AD) were enrolled at baseline. The CN class consists of healthy aging controls with no conversion within 3 years of follow-up visits from baseline. Subjects diagnosed with mild cognitive problems without losing their ability to carry out daily activities were retained in the MCI class. The AD class comprises patients identified as AD through diagnosis at baseline and exhibit no sign of reversion within 2 years of follow-up visits.

All the acquired sMRI were generated from scanners of various manufacturers, such as Philips, Siemens, and General Electric. On account of the various acquisition protocols, the dataset will undergo a preprocessing procedure. There is 1.2 mm spacing between two MRI scans, and the dimension of a voxel is 256 × 256 × 256. In terms of resolution, there is only a slight difference found across the patients. The data used were restricted to the standard 1.5 T T1-weighted sMRI, which were acquired by the volumetric three-dimensional magnetization-prepared rapid gradient-echo (3DMPRAGE) protocol. Other data acquisition settings include 8-channel coil, TR = 650 ms, TE = minimum full, flip-angle = 8°, and FOV = 26 cm. Participants may have multiple scans at baseline and follow-up visits (after 1, 2, and 3 years).

The data used were restricted to the standard 1.5 T T1-weighted sMRI, which were acquired by the volumetric three-dimensional magnetization-prepared rapid gradient-echo (3DMPRAGE) protocol. Other data acquisition settings include 8-channel coil, TR = 650 ms, TE = minimum full, flip-angle = 8°, and FOV = 26 cm. Participants may have multiple scans at baseline and follow-up visits (after 1, 2, and 3 years). It is important to note that not all participants appeared at every planned follow-up visit. Some participants were retained in the study without appearing at every follow-up meeting. There was also a significant decrease in follow-up visit rate after 2 years, indicating that fewer data were available over time. Table 1 summarizes the demographic information for the 819 subjects with age ranges from 55 to 92 years, including 192 patients with AD, 398 subjects belonging to the MCI, and 192 who are cognitively normal and were included in the study. Based on Table 1, it can be seen that the CN group is more educated than the MCI and AD groups with mean education years of 16.0 ± 2.9 years, and the MCI group is the youngest among the three groups with a mean age of 74.7 ± 7.4 years.

**TABLE 1:** Demographic of participants with MCI and AD and cognitive normal subjects from the study population.

Diagnostic type	Number of participants	Age	Gender (M/F)	Education (years)
CN	229	75.8 ± 5.0 (59.9–89.6)	119/110	16.0 ± 2.9 (6–20)
MCI	398	74.7 ± 7.4 (54.5–89.3)	257/141	15.7 ± 3.0 (4–20)
AD	192	75.3 ± 7.5 (55.1–90.9)	101/91	14.7 ± 3.1 (4–20)

### Proposed Model

**FIGURE 1:**
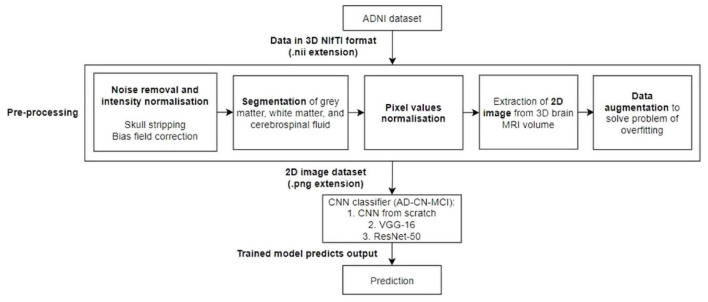
Workflow of the proposed model. A preprocessing steps, which include noise removal and intensity normalization, segmentation, pixel value normalization, and 2D image extraction and data augmentation, will be performed followed by the classification by an AD-CN-MCI CNN classifiers.

The process of the proposed approach is depicted in Figure 1. As a result, the acquired ADNI1 dataset is initially subjected to a number of preprocessing methods. The retrieved 2D images are then divided into training, validation, and testing sets. Three CNN models are evaluated: a CNN trained from scratch, VGG-16, and ResNet-50. The training data was supplemented before feeding the training data into the CNN models for training.

### Preprocessing

Preprocessing was applied to each brain sMRI to normalize the data into desired form and format. The routine of preprocessing steps can be summarized into six different steps: (1) skull-stripping, (2) non-uniform intensity correction, (3) segmentation, (4) extraction of 2D image from 3D MRI volume, (5) pixel values normalization, and (6) data augmentation.

#### Skull Stripping

**FIGURE 2:**
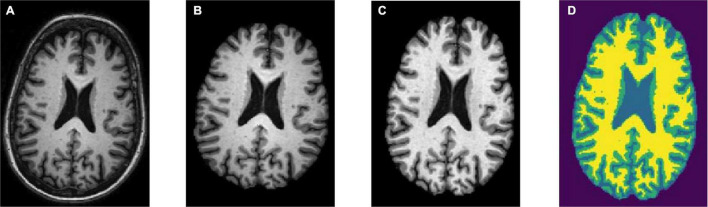
Preprocessing steps: **(A)** raw bran MRI, **(B)** skull stripped MRI, **(C)** bias field corrected MRI, and **(D)** tissue segmented MRI (WM is denoted in yellow, GM is denoted in green, and CSF is denoted in light blue).

Skull stripping is the removal of the skull from a 3D brain MRI. For quantitative morphometric studies, the skull is the non-brain tissue that functions as noise, lowering CNN classification performance (Goceri and Songül, 2017). Aside from that, removing the skull from the brain enhances segmentation outcomes. To obtain solely the brain tissues, the skull section was stripped or deleted using the DeepBrain library. Figures 2A,B depicts the raw brain had its skull stripped together with intensity normalized using the DeepBrain library.

#### Bias Field Correction

Strong bias fields are known to cause voxel tissue type mislabeling, undermining the algorithm’s accuracy, which is based on gray and white matter contrast (Gupta et al., 2019). To keep this impact to a minimum, the N4 bias field correction method was used in conjunction with the SimpleITK library for correcting low-frequency intensity presented non-uniformly in brain sMRI (Tustison et al., 2010). Following that, the intensity variation of the same brain tissue was deleted based on its location within the image. The bias-corrected brain displayed more consistent intensity in the white matter region (Figure 2C).

#### Tissue Segmentation

The hidden Markov random field (HMRF) tissue classifier was used to segment T1-weighted sMRI data that had previously been skull-stripped and bias field corrected (Zhang et al., 2001). The hidden Markov models were used to develop the HMRF idea. In contrast to hidden Markov, HMRF features an underlying Markov random field rather than a Markov chain.

The brain sMRI volumes were segmented into three different regions of GM, WM, and CSF using the HMRF tissue classifier from the DIPY library. These three main features were used to differentiate AD from MCI and CN. Alterations in WM and GM were commonly used for the analysis of AD progression (Klöppel et al., 2008). In ML approach studies, it would be laborious to perform tissue segmentation and feature extraction. Hence, automated segmentation is essential for a dataset with a large number of images. Figure 2D shows the plotting of the resulting segmentation with a clear separation between GM, WM, and CSF.

#### Extraction of 2D Images From the 3D Volume

The Matplotlib library was used to extract 2D slices or images from the segmented 3D MRI after the segmentation phase. More specifically, brain pictures in PNG format were recovered from the axial view of the 3D MRI slices ranging from the 160th to 170th slice. Slices in this range provide a wealth of information about the GM, WM, and CSF. For the three courses, a total of 2,387 brain scans were performed (CN, MCI, and AD). Good model performance is associated with selecting the best available slices containing relevant morphological information (Stoeckel and Fung, 2005). Given the preferable slice range, every interval of five slices (e.g., 160th, 165th, and 170th) of three brain images were extracted from the MRI volume of the AD and CN subjects, in which AD and MCI have 2,043 and 2,051 images, respectively. One scan was removed from the CN class due to file corruption. In addition, two brain images (160th and 165th) were extracted for the MCI class that yielded 2,044 images.

A padding private function was implemented to add padding to all final images, so that the output images have a uniform dimension of 271 × 271 pixels. Here, the images were saved in gray scale format and named according to their classes with a number suffix in an increasing sequence. After preprocessing, the data were all in the form of 2D images. This helps to substantially reduce the dataset size from 37 GB to 260 MB.

#### Pixel Values Normalization

As of this stage, every image data were in gray scale with pixel values ranging between 0 and 255 (8-bit). Before the training process, we normalize every image pixel value with a value between 0 and 1.

#### Data Augmentation

The process of data augmentation was performed to mitigate the general problem of the small dataset, which is overfitting during training, by applying various transformations on the images from the dataset. The transformations used were rotation of 15°, zoom range of 0.10°, height shift range of 0.10°, and width shift range of 0.10°.

### Prediction Model

The CNN models used in this study will be described in detail here. To perform the 3-way classification task, three different CNN models were tested. The first model is a CNN that was trained from the ground up. Furthermore, the second and third models used the transfer learning technique. CNN models with pretrained ImageNet weights, such as VGG-16 and ResNet-50, were used instead of training a model from scratch. These models were trained to classify 1,000 different image classes using the ImageNet database, which contains over a million images.

#### Convolutional Neural Network From Scratch

**FIGURE 3:**
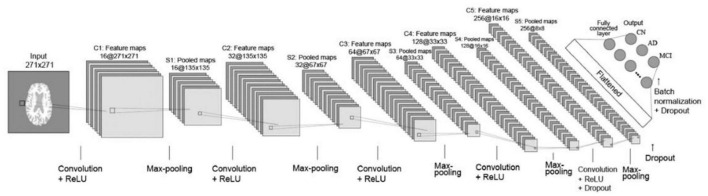
Layout of CNN trained from scratch. Briefly, the architecture comprises the following: 5 convolutional layers followed by ReLU activation; 5 layers of max-pooling layers; 2 dropout layers; a flatten layer; a fully connected layer with 256 neurons followed by a dropout layer and a batch normalization layer; and ultimately an output layer with softmax activation, which outputs the probability of prediction for each class.

Figure 3 depicts the 2D CNN architecture that was created from scratch. In a nutshell, the architecture consists of the following elements: five convolutional layers followed by ReLU activation; five max-pooling layers; two dropout layers; a flatten layer; a fully connected layer with 256 neurons followed by a dropout layer and a batch normalization layer; and, finally, an output layer with softmax activation that outputs the probability of prediction for each class.

The CNN first layer was fed preprocessed axial view brain sMRI data. The second layer was a convolutional layer that performed convolution operations on input images and filtered the output to produce multiple feature maps. There were five convolution layers in total, each with 16-32-64-128-256 feature maps. All of the convolution filters had a size of 22, a stride of one, and “same” padding, which ensured that the output was the same size as the input. After each convolutional layer, a max-pooling layer with 22 regions was applied. The pooling layers functioned as down-sampling layers, resulting in the creation of multiple pooled maps. The final two pooling layers were followed by a dropout layer with a dropout rate of 0.5, which meant that 50% of the nodes in the layers would be dropped out to ensure regularization and prevent overfitting. The pooled feature maps were then flattened to a 1D vector and fed into the next fully connected layer with 256 neurons. A batch normalization layer was added before the dropout layer to improve the model’s regularization even further. The final layer is the output layer with three nodes incorporating softmax activation function to determine the probabilities of each possible class of the classification task. Finally, a vector consisting of probabilities belonging to the AD, CN, and MCI classes was obtained as the final classification result.

#### VGG-16

In this study, the pretrained VGG-16 model was used in the form of a feature extractor (Simonyan and Zisserman, 2015). Also, VGG-16 with pretrained weights was used as a bootstrap feature extractor for feature extraction from the preprocessed brain sMRI images. The extracted features were then directed to a new classifier, which was trained from scratch.

It is important to note that the gray scale image dataset could not be directly fed to the VGG-16 model because it is a pretrained model with a fixed input configuration. VGG-16 requires RGB images with three channels as input. A gray scale image, on the contrary, has only one channel. The obvious solution is to iteratively repeat all of the image arrays in the dataset three times on a new dimension. As a result, the same image would appear in all three channels. This was accomplished by specifying the color mode as “RGB” in the Keras library’s flow from directory method.

#### ResNet-50

The pretrained ResNet-50 model was used as a feature extractor, similar to VGG-16, and a new densely connected classifier was used for prediction (He et al., 2016). Deep neural network training is difficult because adding more layers causes the infamous vanishing gradient problem, also known as the exploding gradient problem. The main feature of ResNet is the design of residual connections. The residual block enabled ResNet to connect the previous layer to the current layer as well as the layer behind the previous layer. As a result, each layer can capture more than just the observations of the previous layer. Furthermore, the batch normalization layer is placed after each convolutional layer in ResNet. Batch normalizations normalize layer weights, allowing for faster training rates. This speeds up deep network training and reduces the vanishing gradient problem.

#### Parameters and Evaluation Metrics

Table 2 summarizes the best parameter combinations for training the three networks. In addition, the evaluation metrics were accuracy, precision, recall, and F1-score. Keras, an open-source high-level neural network API for building deep models, was used to build all of the deep learning models, with TensorFlow as the backend. Keras was chosen because it enables rapid prototyping and parallel computing with GPUs. In this study, training, validation, and testing routines were carried out on Google Colab in order to execute Python 3 codes for data preprocessing and the development of a CNN model. The GPU model would be assigned at random based on the availability on Google Colab. There was no published limit on the idle timeout period, RAM size, or disc size. Typically, a RAM size of around 13 GB and a disc size of around 70 GB would be allocated for GPU accelerated runtime.

**TABLE 2:** Hyperparameters of CNNs adopted in the experiments.

Parameter	CNN	VGG-16	ResNet50
Number of epochs	100	100	100
Batch size	512	256	256
Weight initializer	Xavier uniform	Xavier uniform	Xavier uniform
Optimizer	Adam	Adam	Adam
Adam parameters	β1 = 0.9, β2 = 0.999	β1 = 0.9, β2 = 0.999	β1 = 0.9, β2 = 0.999
Learning rate	10-4	10-5	10-4
Loss function	Categorical cross-entropy	Categorical cross-entropy	Categorical cross-entropy
Metrics	Accuracy	Accuracy	Accuracy
Data augmentation	Rotation, zoom, height shift, width shift, shear, horizontal flip	Rotation, zoom, height shift, width shift, shear, horizontal flip	Rotation, zoom, height shift, width shift, shear, horizontal flip

*All the architectures adopted Xavier’s uniform as the weight initializer and Adam as the optimizer.*

In addition, to facilitate model training, two types of “callbacks” in Keras were implemented during training: Early Stop and ModelCheckpoint. Early Stop enabled the models to stop training if their performance did not improve after five epochs of monitoring validation loss. This is one method for preventing a model from overfitting. Next, ModelCheckpoint ensured that models always saved the best weights while training to avoid loss of progression. Saving the weights is more efficient than saving the entire model’s information because a large network like VGG-16 can take up at least 500 MB of memory.

## Results and Discussion

### Training and Validation Performance

Table 3 reports the training and validation performance of the three different CNN models being experimented.

**TABLE 3:** Summary of training and validation performance.

Model	Training time (minutes)	Steps	Training		Validation	
			Accuracy	Loss	Accuracy	Loss
CNN	46	97	0.8755	0.3102	0.7270	0.7094
VGG-16	75	57	0.9492	0.1511	0.8066	0.5263
ResNet-50	91	56	0.9164	0.2150	0.7686	0.5901

To avoid overfitting, all model training was completed with an early stop and a patience level of 15 epochs. The training and validation routines were halted when the validation loss began to deteriorate. The CNN trained from scratch finished training in 46 min, making it the quickest of the three models. Deep CNN, such as VGG-16 and ResNet-50, with multiple stacking layers, can be computationally expensive and take much longer to train than a shallow model trained from scratch. ResNet-50’s longer training time can be attributed to a large number of trainable parameters. The ResNet-50 model, which has a frozen convolutional base and a swapped densely connected classifier, has 42.5 million trainable parameters.

The VGG-16 model, on the contrary, had an identical densely connected classifier and a frozen convolutional base with 8.4 million trainable parameters. Interestingly, despite having five times the number of trainable parameters as VGG-16, ResNet-50 spent only 21.33% more time on training. The inclusion of multiple batch normalization layers between the convolutional layer and the non-linear activation function may be the primary reason for this, allowing a higher learning rate to be used (He et al., 2016).

The loss function quantifies a model’s performance in classifying input images from a dataset. The loss value indicates how well a model performs after each optimization epoch. The goal of training a deep learning network is to minimize the error calculated using the loss function while increasing testing accuracy. VGG-16 achieved a training loss value of 0.1511, while CNN from scratch and ResNet-50 achieved training loss values of 0.3102 and 0.2150, respectively. In terms of validation performance as measured by loss value, VGG-16 achieved the lowest loss value of 0.5263. ResNet-50 came in second with a loss value of 0.5901, and CNN from scratch came in third with a loss value of 0.7094.

The transfer learning method was tested for its ability to produce satisfactory results on small datasets, as seen in recent literature. Deep models with pretrained weights, such as VGG-16 and ResNet-50, were used for feature extraction instead of learning the convolutional bases from scratch. To improve the output classification scores, a new densely connected classifier trained from scratch was added to both models. Both VGG-16 and ResNet-50 outperform the CNN trained from scratch in this case. Despite the use of various regularization methods, such as dropout, batch normalization, and data augmentation, the overfitting problem persists in both models.

### Testing Performance and Discussion

**FIGURE 4:**
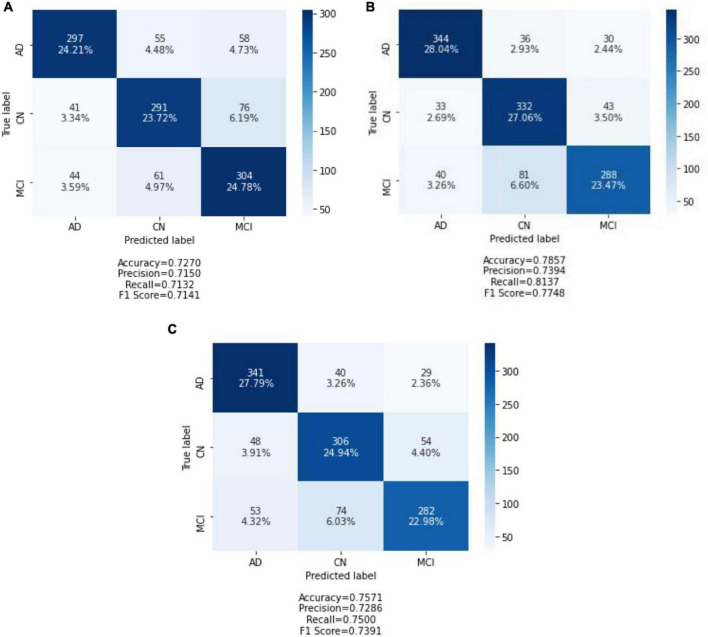
Confusion matrix of three models on test data: **(A)** CNN from scratch; **(B)** VGG-16; and **(C)** ResNet-50. Each of the confusion matrices is visualized as a color-coded heat map using the seaborne library. It can be observed that all the plotted confusion matrices have darker cells for the diagonal elements. This indicates that more data are being predicted correctly to their respective label. Conversely, the off-diagonal elements with light shades indicate misclassifications done by the model.

After all of the models had been trained and validated, the 20% held out testing data were run on each and every model. The confusion matrix was used as a tool to assess model classification performance, along with a summary of prediction results. The number of correctly or incorrectly predicted predictions is summarized systematically in a table, with count values broken down by class. The confusion matrix is a table with three rows and three columns because it is a three-way classification task with three different classes. The predicted lab is represented by the rows (*y*-axis), and the predicted label is represented by the columns (*x*-axis). Figure 4 depicts confusion matrices that describe each model’s classification performance on test data. Using the seaborne library, each confusion matrix is visualized as a color-coded heat map. The darker cells for the diagonal elements can be seen in all of the plotted confusion matrices. This indicates that a large amount of data is correctly predicted according to its label. The off-diagonal elements with light shades, on the contrary, indicate model misclassifications.

The CNN predicted the MCI group with the highest accuracy and the CN group with the lowest accuracy when trained from scratch. It correctly classified 304 of 409 MCI images and 291 of 408 CN images. In contrast, the AD group has the highest classification accuracy in VGG-16 and ResNet-50, while the MCI group has the lowest classification accuracy. ResNet-50 classified 341 AD images out of 410 AD images predicted by VGG-16. In the MCI group, VGG-16 correctly predicted 288 images, and ResNet-50 correctly classified 282 of 409 AD images.

**FIGURE 5:**
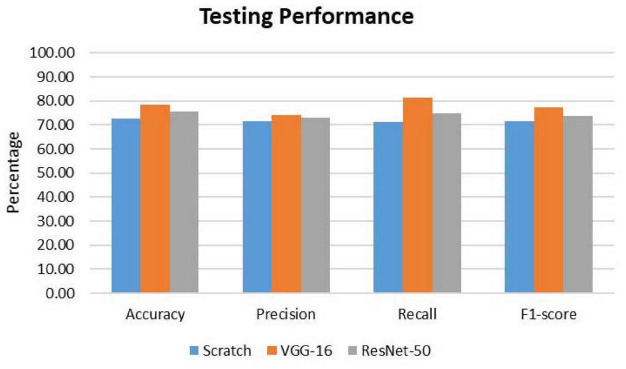
Comparison of classification performance on test data. For all the metrics, VGG-16 ranks the highest.

To further evaluate the classification model, classification metrics such as accuracy, precision, recall, and F1-score were calculated with the aid of the confusion matrices. For each classification model (CNN from scratch, VGG-16, and ResNet-50), the reported classification performance on test data is accuracy of 72.70, 78.57, and 75.71%, respectively; precision of 71.50, 73.94, and 72.86%, respectively; recall of 71.32, 81.37, and 75.00%, respectively; and F1-score of 71.41, 77.48, and 73.91%, respectively. Based on Figure 5, it is observed that VGG-16, which achieved the lowest loss value of 0.5263, performed the best on test data with an accuracy of 78.57%. The lowest testing accuracy of 72.70% is obtained using the CNN from scratch.

For further in-depth evaluation of performance on test data, the classification results for each class label are reported in Table 4. Similar to what was being analyzed using the confusion matrices, the AD group has the highest accuracy value for VGG-16 and ResNet-50. VGG-16 performed the greatest in predicting AD class with an accuracy of 83.90%, precision of 82.49%, recall of 83.90%, and F1-score of 83.19%. Interestingly, ResNet-50 has the lowest accuracy in predicting the MCI class. Overall, using VGG-16 improved the performance values for all three classes.

**TABLE 4:** Testing accuracy, precision, recall, and F1-score for all class label.

Model	Class label	Accuracy	Precision	Recall	F1-score
CNN from scratch	AD	0.7244	0.7775	0.7244	0.7500
	CN	0.7132	0.7150	0.7132	0.7141
	MCI	0.7433	0.6941	0.7433	0.7178
VGG-16	AD	**0.8390**	**0.8249**	**0.8390**	**0.8319**
	CN	0.8137	0.7394	0.8137	0.7748
	MCI	0.7042	0.7978	0.7042	0.7481
ResNet-50	AD	0.8317	0.7715	0.8317	0.8005
	CN	0.7500	0.7286	0.7500	0.7391
	MCI	0.6895	0.7726	0.6895	0.7287

*Bold values are highest value.*

The AD group scored the highest accuracy value for VGG-16 and ResNet-50. VGG-16 performed the greatest in predicting AD class with an accuracy of 83.90%, precision of 82.49%, recall of 83.90%, and F1-score of 83.19%.

## Discussion

From the results obtained, the VGG-16 model outperformed the CNN trained from scratch and the ResNet-50 model. It has the best testing performance with an accuracy of 78.57%, precision of 73.94%, recall of 81.37%, and F1-score of 77.48%. Comparing its performance to other related works, VGG-16 has a performance below the average. Being trained on the ImageNet dataset, VGG-16 was able to extract representations using its convolutional base for learning the multiclass classification task. Despite the great performance on learning the representations, VGG-16 still encountered the typical overfitting problem due to the small dataset used. Several regularization methods were used, such as dropout, batch normalization, data augmentation, and early stopping. However, the signs of overfitting can still be noticed. This could be due to the high complexity of the classification task. The subtle discrepancies between the MCI and AD images require a large amount of data to learn the representation to classify them. With the small dataset being used in this project, the VGG-16 model could not learn the problem completely, hence the overfitting problem. Another possible reason could be that the dataset being used in this project has substantial differences as compared to the ImageNet dataset. The VGG-16 was pretrained on general images from the ImageNet without including medical images. Hence, the high-level features learned by the higher layers of the VGG-16 are not sufficient to differentiate the classes in this study.

Based on the results obtained, it is of importance to choose a proper training strategy for the model. Hence, the model is able to spend the least time training while trying to cover as many cases as possible. An adequate model capacity is essential for model generalization. Model depth should be kept as small as possible to prevent a model from overfitting on training data. The greater the depth, the more cases the model can memorize. As a consequence, the final system will perform worse on unseen data. Another possible reason behind inferior performance could be insufficient data augmentation. The data augmentation used is insufficient to generate diversity for the original dataset. An example of aggressive data augmentation can be seen in the study by Basaia et al. (2019). Apart from general augmentation transformations such as rotation, zooming, and scaling, the study implemented deformation, cropping, and flipping.

The strengths of this study are elaborated as follows. In general, most of the studies emphasized performing binary classification of different phenotypes of AD. In this study, three different classes (AD, CN, and MCI) are classified directly using a single classifier. This study is less common as most of the studies deal with the problem of multiple class labels by dividing the problem into several binary sub-problems. Moreover, tissue-segmented sMRI brain images were used, which substantially lower the requirement for computational costs in terms of power and time. Second, MRI images were segmented into GM, WM, and CSF for training and testing the model. Moreover, models were tested using an independent set of images held out from the dataset. In addition, the performance of popular deep transfer learning models such as VGG-16 and ResNet-50 was evaluated to study their performance on images not from the ImageNet domain.

## Conclusion

In this study, we have conducted a series of experiments with different deep learning CNN architectures on preprocessed axial sMRI brain images retrieved from the ADNI database. To address the problem of classifying brain sMRI images of three distinct classes of AD, CN, and MCI, three different CNN models were built, namely, a CNN from scratch, VGG-16, and ResNet-50. The VGG-16 model outperforms the other two models in testing. The results show that, despite being trained on general images from the ImageNet dataset, VGG-16 is capable of extracting relevant features for the classification task. Using the same dataset, the pretrained VGG-16 outperforms shallow CNN and classical machine learning algorithms. However, its performance is considered subpar when compared to other literature, which also employed deep learning techniques. Increasing the number of data for training is the main factor for improving classification performance. This project serves as a catalyst to motivate further study on computer-assisted AD diagnosis systems that can provide automated early diagnosis of AD and the detection of more phenotypes of AD.

For future studies, a list of improvements can be suggested. Effort should be devoted in attempting different pretrained CNN families such as AlexNet (Krizhevsky, 2014), Xception (Chollet, 2017), Inception (Szegedy et al., 2016), MobileNet (Howard et al., 2017), and other variants of VGG and ResNet as well as the more recent state-of-the-art network (Tan and Le, 2019, 2021) as base model for feature extraction. Furthermore, classification performance could be improved through fine-tuning. Unfreeze some layers, or even half of the model, for training with the classifier at a slower learning rate. Finally, a few different methodologies for improving classification performance to distinguish between AD, CN, and MCI may be investigated in the future. One method is to include multimodal data in the study. Multimodal research necessitates feature fusion to combine features from various modalities into a single feature vector. Another method for improving performance is to enrich the feature learning process by fusing low-dimensional features like clinical scores with the MRI features space.

## Data Availability Statement

The data were available from the database of the Alzheimer’s Disease Neuroimaging Initiative (ADNI), which is available publicly upon approval from the ADNI.

## Author Contributions

All authors listed have made a substantial, direct, and intellectual contribution to the work, and approved it for publication.

## Conflict of Interest

The authors declare that the research was conducted in the absence of any commercial or financial relationships that could be construed as a potential conflict of interest.

## Publisher’s Note

All claims expressed in this article are solely those of the authors and do not necessarily represent those of their affiliated organizations, or those of the publisher, the editors and the reviewers. Any product that may be evaluated in this article, or claim that may be made by its manufacturer, is not guaranteed or endorsed by the publisher.
